# Time Trends and Variation in the Use of Active Surveillance for Management of Low-risk Prostate Cancer in the US

**DOI:** 10.1001/jamanetworkopen.2023.1439

**Published:** 2023-03-02

**Authors:** Matthew R. Cooperberg, William Meeks, Raymond Fang, Franklin D. Gaylis, William J. Catalona, Danil V. Makarov

**Affiliations:** 1 Department of Urology, UCSF Helen Diller Family Comprehensive Cancer Center, San Francisco, California; 2 Department of Epidemiology and Biostatistics, UCSF Helen Diller Family Comprehensive Cancer Center, San Francisco, California; 3 American Urological Association Education and Research Inc, Linthicum, Maryland; 4 Genesis Health Care Partners, San Diego, California; 5 Department of Urology, Northwestern University Feinberg School of Medicine, Chicago, Illinois; 6 Department of Urology, New York University, New York, New York

## Abstract

**Importance:**

Active surveillance (AS) is endorsed by clinical guidelines as the preferred management strategy for low-risk prostate cancer, but its use in contemporary clinical practice remains incompletely defined.

**Objective:**

To characterize trends over time and practice- and practitioner-level variation in the use of AS in a large, national disease registry.

**Design, Setting, and Participants:**

This retrospective analysis of a prospective cohort study included men with low-risk prostate cancer, defined as prostate-specific antigen (PSA) less than 10 ng/mL, Gleason grade group 1, and clinical stage T1c or T2a, newly diagnosed between January 1, 2014, and June 1, 2021. Patients were identified in the American Urological Association (AUA) Quality (AQUA) Registry, a large quality reporting registry including data from 1945 urology practitioners at 349 practices across 48 US states and territories, comprising more than 8.5 million unique patients. Data are collected automatically from electronic health record systems at participating practices.

**Exposures:**

Exposures of interest included patient age, race, and PSA level, as well as urology practice and individual urology practitioners.

**Main Outcomes and Measures:**

The outcome of interest was the use of AS as primary treatment. Treatment was determined through analysis of electronic health record structured and unstructured clinical data and determination of surveillance based on follow-up testing with at least 1 PSA level remaining greater than 1.0 ng/mL.

**Results:**

A total of 20 809 patients in AQUA were diagnosed with low-risk prostate cancer and had known primary treatment. The median age was 65 (IQR, 59-70) years; 31 (0.1%) were American Indian or Alaska Native; 148 (0.7%) were Asian or Pacific Islander; 1855 (8.9%) were Black; 8351 (40.1%) were White; 169 (0.8%) were of other race or ethnicity; and 10 255 (49.3%) were missing information on race or ethnicity. Rates of AS increased sharply and consistently from 26.5% in 2014 to 59.6% in 2021. However, use of AS varied from 4.0% to 78.0% at the urology practice level and from 0% to 100% at the practitioner level. On multivariable analysis, year of diagnosis was the variable most strongly associated with AS; age, race, and PSA value at diagnosis were all also associated with odds of surveillance.

**Conclusions and Relevance:**

This cohort study of AS rates in the AQUA Registry found that national, community-based rates of AS have increased but remain suboptimal, and wide variation persists across practices and practitioners. Continued progress on this critical quality indicator is essential to minimize overtreatment of low-risk prostate cancer and by extension to improve the benefit-to-harm ratio of national prostate cancer early detection efforts.

## Key Points

**Question:**

What are the recent trends and ongoing variation in the use of active surveillance for patients diagnosed with low-risk prostate cancer, as determined using the large, national American Urological Association Quality Registry?

**Findings:**

In a cohort study of more than 20 000 men treated at nearly 350 urology practices across the US, with data drawn directly from electronic health record systems, rates of active surveillance increased sharply from 26.5% in 2014 to 59.6% in 2021. However, the use of surveillance varied widely across practices and individual urology practitioners.

**Meaning:**

These findings suggest that active surveillance rates are rising nationally but are still suboptimal, and local variation is high; improving these practice patterns is essential to improve our national prostate cancer outcomes.

## Teaser

This cohort study of men treated at US urology practices uses a national disease registry to analyze trends and variation in the use of active surveillance for the treatment of low-risk prostate cancer.

## Introduction

Prostate cancer is the second leading cause of cancer mortality among US men,^1^ yet most cancers identified through screening efforts are relatively indolent, posing minimal threat to length or quality of life for years or decades after diagnosis. Early detection and treatment of aggressive cancers have helped decrease mortality rates by half in the prostate-specific antigen (PSA) era^1^ but at the cost of extensive, avoidable morbidity and cost attributable to overtreatment of indolent tumors. Prostate cancer risk can be reliably classified using well-validated, accurate tools that have been available for more than 20 years,^2,3^ but historically, prostate cancers have been treated regardless of risk.

Active surveillance (AS) for prostate cancer aims to avoid or at least delay the potential complications and adverse effects of treatment while maintaining expectation of cure if early signs of progression are identified. Active surveillance is now endorsed as the preferred management for low-risk prostate cancer by all major clinical guidelines. National AS rates historically have been low outside academic urology settings but recently have been increasing rapidly.^4,5,6^ Existing analyses, however, have been restricted to specific geographic areas or clinical contexts, face long lag times to data access, and have been able to examine variations in practice to a limited extent. We analyzed AS trends, associations, and variation in the American Urological Association (AUA) Quality (AQUA) Registry, comprising near real-time data collected directly from urology practices nationwide.

## Methods

The AUA launched the AQUA Registry in 2013 to help urology practices understand and improve their quality of care and to streamline quality reporting. Data are accessioned directly from a variety of electronic health record (EHR) systems at participating practices. Structured data are collected directly, and other data (eg, grade and stage) are identified via regular expression searches and natural language processing.^7^ Race data are collected as documented in the EHR demographic characteristics tables and matched to US Census categories: American Indian or Alaska Native, Asian or Pacific Islander, Black (including African American), and White (including Caucasian). Other racial categories, including multiracial, are included as Other. The AQUA Registry is supervised by a central institutional review board (Advarra), which allows and governs retrospective analysis of deidentified data for research purposes; as such, informed consent was waived for this study. We followed the Strengthening the Reporting of Observational Studies in Epidemiology (STROBE) guidelines in conducting and reporting this study.

The AQUA Registry comprised more than 8.5 million unique patients, including 298 081 ever treated for prostate cancer from January 1, 2014, to June 1, 2021. Low risk was defined as a PSA level less than 10 ng/mL, grade group (GG) 1, and clinical stage T1/T2a. Patients were also included as having low-risk disease if information on stage was missing and PSA level and GG both qualified for low risk. Primary treatments were classified as radical prostatectomy, external beam radiation (EBRT), brachytherapy, androgen deprivation monotherapy, or AS. Focal therapy was uncommon and inconsistently documented and so was not included in this analysis. For practices that do not offer EBRT within the practice, coding data cannot identify EBRT, and documentation quality is variable. Therefore, practices with no EBRT cases (n = 7) were excluded entirely. Determination of AS was made via natural language processing of clinical records and analysis of billing codes; if primary treatment was not clear from the analysis of notes, AS was defined by the absence of any active treatment and, to distinguish AS from loss to follow-up, by at least 1 subsequent PSA level within the first year that remained greater than 1.0 ng/mL.

### Statistical Analysis

Descriptive statistics were calculated and demographic and clinical variables were compared between AS and non-AS patients via unpaired, 2-tailed *t* tests and χ^2^ tests, as appropriate. Given conflicting prior reports on variation by race in use of AS,^6,8,9^ use of primary treatments was examined over time for all men and separately for Black and White men. Variation in use of AS was determined based on the proportion of men with low-risk disease managed with AS for each practice and each practitioner, restricted to practices that managed low-risk disease among at least 50 men. These rates were plotted with a linear regression smoother, and correlation between practice- and practitioner-level rates were calculated.

To explore factors associated with the use of surveillance, a hierarchical mixed-effects binominal model was fit, which included as random effects urology practitioner nested within urology practice. Fixed effects included year of diagnosis, age decile at diagnosis, race, PSA at diagnosis, practice volume, and practitioner volume. Urologist, radiation oncologist, total practitioner density, and hospital bed density per capita at the county level for each urology practice were also determined from the US Department of Health and Human Services Area Health Resource File and included in the model as additional fixed effects. Intraclass correlation was calculated to determine the proportion of variation attributable to practice and practitioner. All analyses were performed using R software, version 4.1.2 (R Foundation for Statistical Computing). A 2-sided *P* < .05 was considered to be statistically significant.

## Results

By the middle of 2021, a total of 1945 urology practitioners, representing 349 practices in 48 US states and territories (eFigure 1 in Supplement 1), were included in the AQUA Registry. Among these practices, mean (SD) practitioner count was 9 (19). Of the practices, 78 were solo practices, 83 had 2 to 5 practitioners, and the larger practices ranged up to 251 practitioners. Among 298 801 patients newly diagnosed with prostate cancer in the AQUA Registry from 2014 to 2021, 27 289 were newly diagnosed with low-risk disease. Of these, 20 809 (76.3%) had known primary treatment (eFigure 2 in Supplement 1). Of the 20 809 patients with known primary treatment, 31 (0.1%) were American Indian or Alaska Native, 148 (0.7%) were Asian or Pacific Islander, 1855 (8.9%) were Black, 8351 (40.1%) were White, 169 (0.8%) were of other race or ethnicity, and 10 255 (49.3%) were missing information on race or ethnicity. The median age at diagnosis was 65 (IQR, 59-70) years. Other characteristics are summarized in the Table; compared with immediately treated patients, AS patients generally were older, had their cancer diagnosed more recently, and were slightly more likely to have clinical stage T1 rather than T2 disease. Over time, the proportion of all patients with newly diagnosed low-risk disease varied only slightly, between 17.4% and 19.3% per year, with no discernible trend over time. Practice volume for low-risk disease across the study period ranged from 1 to 2560 unique patients. For individual practitioners, the range was 0 to 169.

**Table.** Characteristics of the Study Patients^a^

Characteristic	Active treatment (n = 11 840)	AS/WW (n = 8969)	*P* value
Race			
American Indian or Alaska Native	17 (0.1)	14 (0.2)	.11
Asian or Pacific Islander	76 (0.6)	72 (0.8)	
Black	1125 (9.5)	730 (8.1)	
White	5029 (42.5)	3322 (37.0)	
Other^b^	92 (0.8)	77 (0.9)	
Missing	5501 (46.5)	4754 (53.0)	
Age decile, y			
40s	508 (4.3)	261 (2.9)	<.001
50s	3190 (26.9)	2189 (24.4)	
60s	5506 (46.5)	4263 (47.5)	
70s	2373 (20.0)	2059 (23.0)	
80s	256 (2.2)	193 (2.2)	
Missing	7 (0.1)	4 (0.0)	
Year of diagnosis			
2014	1406 (11.9)	506 (5.6)	<.001
2015	1466 (12.4)	692 (7.7)	
2016	1738 (14.7)	812 (9.1)	
2017	1756 (14.8)	967 (10.8)	
2018	1677 (14.2)	1250 (13.9)	
2019	1650 (13.9)	1747 (19.5)	
2020	1383 (11.7)	1870 (20.8)	
2021	764 (6.5)	1125 (12.5)	
PSA level at diagnosis, ng/mL			
Mean (SD)	5.31 (1.97)	5.31 (1.89)	.96
Median (range)	5.10 (0.60-10.00)	5.09 (1.00-10.00)	
T stage			
T1	3506 (29.6)	2354 (26.2)	<.001
T2	984 (8.3)	490 (5.5)	
Missing	7350 (62.1)	6125 (68.3)	

Abbreviations: AS/WW, active surveillance or watchful waiting; PSA, prostate-specific antigen.

SI conversion factors: To convert PSA to micrograms per liter, multiply by 1.

^a^ Data are presented as number (percentage) of patients unless otherwise indicated.

^b^ Other race includes any identified race in the electronic health record that did not match 1 of the US Census categories, including multiracial.

**Figure 1.**
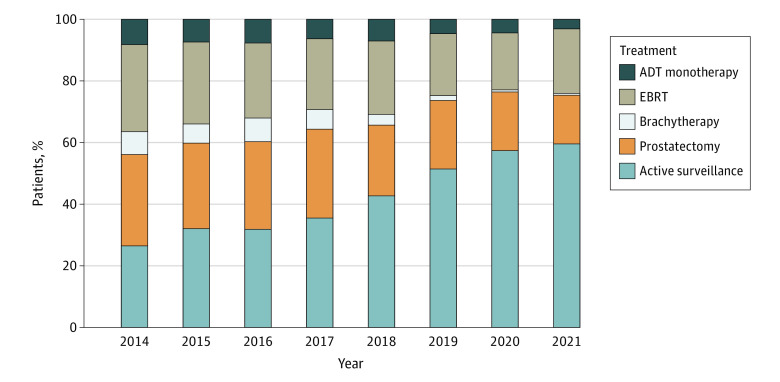
Treatment of Low-risk Prostate Cancer Over Time ADT indicates androgen deprivation therapy; EBRT, external-beam radiation therapy.

**Figure 2.**
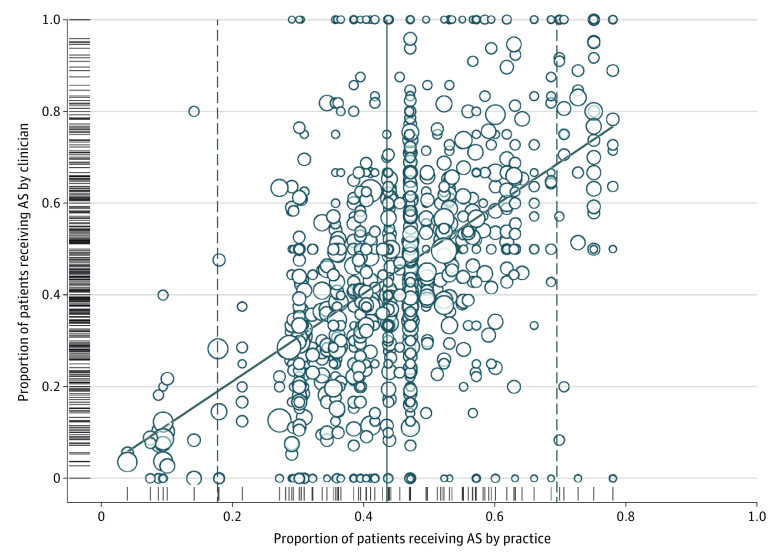
Variation in Use of Active Surveillance (AS) Among Men With Low-risk Prostate Cancer The circles represent the percentage of patients with low-risk disease managed with AS by each practitioner within each practice with at least 50 low-risk cases managed. Distributions for each level individually (practice and practitioner) are additionally illustrated with hashmarks along the axes. Circle size is proportional to the volume of low-risk prostate cancers managed. The solid and dashed lines indicate the mean and 2 × SDs.

Limiting the analysis to practices with at least 50 low-risk patients, AS rates have more than doubled over time, from 26.5% in 2014 to 59.6% for the first half of 2021 (Figure 1). Overall, AS rates were not meaningfully different between Black (730 of 1855 [39.4%]) and White (3322 of 8351 [39.8%]) patients but were higher for patients of other races or with missing race (4917 of 10 603 [46.4%]). Trends over time were similar for Black and White men (eFigure 3 in Supplement 1). The proportion of patients with low-risk disease managed with AS ranged from 4% to 78% at the practice level and from 0% to 100% at the practitioner level (Figure 2). The linear regression smoother associating practice- and practitioner-level variation approaches the identity line; however, correlation between the 2 was modest (*r* = 0.57, *P* < .001), and the practitioner-level use of AS varied widely within practices with both low and high overall use of AS.

**Figure 3.**
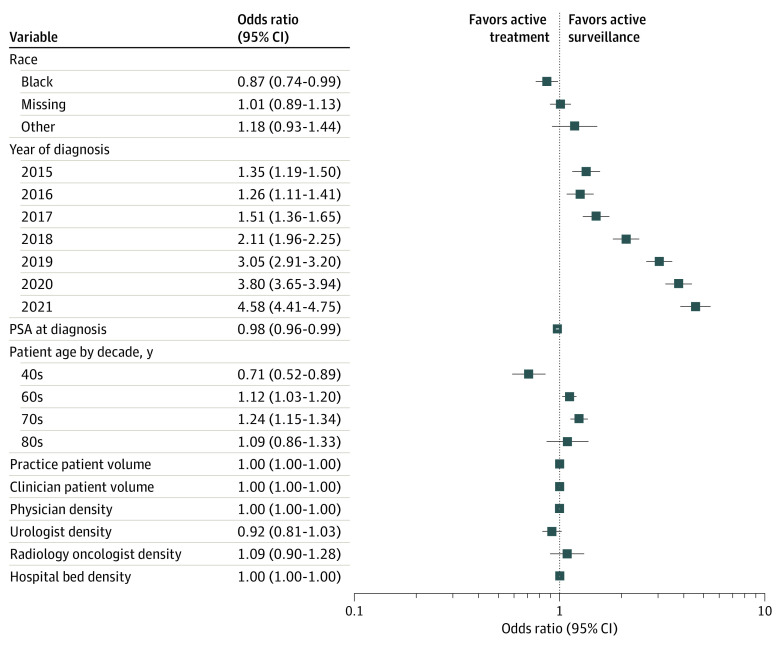
Factors Associated With Active Surveillance The forest plot indicates odds ratios with 95% CIs for each factor in a hierarchical regression model that included practice and practitioner as random effects. Reference groups for multilevel variables are White for race, diagnosed in 2014 for year, and 50s for age decile. PSA indicates prostate-specific antigen.

On hierarchical logistic regression (Figure 3), year of diagnosis was by far the variable most strongly associated with AS use, with an odds ratio (OR) per year of 1.25 (95% CI, 1.24-1.27). The ORs for individual years increased progressively to 4.48 (95% CI, 4.31-4.65) for 2021 relative to 2014. Older patient age and lower PSA level were also associated with AS. Black men were less likely to receive AS than White men (OR, 0.87; 95% CI, 0.75-1.00); other races were not associated with AS rates on multivariable analysis. Repeating the analysis excluding men with missing race did not change any parameter estimates meaningfully, although the OR for Black race, at 0.88 (95% CI, 0.75-1.01), now included 1.0. The intraclass correlation was 0.098 for practitioner and 0.104 for practice. Neither practice volume nor indicators of practitioner density affected the odds of AS. Higher urologist density per capita was associated with lower odds of AS, although not to a statistically significant degree (OR, 0.92; 95% CI, 0.81-1.03).

## Discussion

Although academic series have demonstrated the safety and appropriateness of AS for many years,^10^ its adoption in community practice in the US, for a variety of reasons, has been slow. Until 2010, the proportion of low-risk disease managed with AS in community practice was consistently less than 10%^4,11^; during the past decade, this proportion has improved but remained less than 50%.^6^ The optimal rate of AS has not been defined, but it is likely greater than 80%, a rate that has been reported from the US Veteran Affairs system^12^ and in other health systems.^13^

In this analysis, we report contemporary trends in a large set of patients with low-risk disease, representing practice patterns for highly diverse community urology practices. Rates of AS nationally continue to increase rapidly, but overtreatment of low-risk disease remains excessive. Moreover, individual practices vary radically in their use of AS, as do individual practitioners even within a given practice. Such variation has been identified before but across much smaller sets of practices.^4,14^ This problem is not unique to AS for prostate cancer; small area variations characterize every area of clinical medicine in which multiple treatment options exist.^15^ A recent analysis^16^ from the Michigan Urological Surgery Improvement Collaborative found that with focused attention to use of AS as a quality measure and implementation of a statewide roadmap initiative, rates of AS statewide for low-risk disease increased to approximately 75% but have plateaued in recent years. We found lower odds of AS for Black men on multivariable analysis despite similar overall rates; other data sets have not documented this disparity.^6^ Although some analyses^17^ have documented higher rates of progression for Black men on AS compared with White men, most have not, and guidelines support AS—perhaps at increased intensity of surveillance—for Black men.^18^ Practice and practitioner volume did not affect AS use, nor did most indicators of practitioner density. However, higher urologist density appeared to be associated with somewhat lower odds of AS, suggesting that more competition in a given area tends to create more opportunities for overtreatment.

Prostate-specific antigen–based prostate cancer early detection efforts that aim to identify high-risk, potentially lethal cancers still in the window of opportunity for cure have helped decrease age-adjusted mortality rates by nearly half since the 1990s. However, the principal downside to screening is overdiagnosis and subsequent overtreatment of low-risk disease, the latter associated with long-term potential adverse effects. Active surveillance aims to avoid or defer these effects through monitoring via periodic PSA testing, imaging, and biopsy assessments, with treatment undertaken with curative intent if progressive disease is identified. Routine use of AS for low-risk disease is essential to maintain a favorable benefit-risk ratio for screening. In fact, improving rates of AS were cited by the US Preventive Services Task Force in its 2018 revision of its prostate cancer screening guideline, with PSA screening now upgraded to a “C,” favoring shared decision-making.^19^

Contemporary screening relies increasingly on imaging and biomarkers as secondary tests for men with elevated PSA levels to help determine who should proceed to biopsy. These tests generally dichotomize results as GG2 or higher (positive) vs benign or GG1 (negative), and their increasing use reflects an implicit determination that GG1 does not have to be found. In fact, the proportion of newly diagnosed cancers we identified as low risk—consistently less than 20%—is much lower than the nearly 50% proportions that prevailed in the 2000s.^20^ Autopsy series have shown that histologic GG1 is virtually a normal feature of aging, affecting half of all men by their 70s or 80s,^21^ and the question has been raised whether GG1 should even be called cancer.^22^ Regardless of its label, if GG1 is effectively an unintentional finding, incidental to the search for potentially lethal disease, it should rarely be treated immediately.

### Limitations

This study has some important limitations. Electronic health record data are often incomplete. Race data are self-reported and frequently missing, and ethnicity data are not routinely available. These problems reflect both inconsistent capture of these variables in the EHRs and the inadequacy of census-defined race categories in reflecting complex social determinants of health. Our access to social determinants data for patients in the AQUA Registry is limited, and findings with respect to racial disparities in AS use here and in similar analysis should be interpreted carefully. The AQUA Registry captures radiation therapy data imperfectly, and the quality of AS has not yet been documented; some patients included may be undergoing watchful waiting or may have delayed treatment decision-making. Our definition of AS is intentionally liberal; follow-up with PSA testing alone without subsequent biopsies is not considered adequate AS but has been documented very commonly in community practice.^23^ Future analyses will examine use of PSA testing, imaging, and follow-up biopsy among AS patients in the AQUA Registry. Our use of the 1.0-ng/mL threshold to rule out treated patients is admittedly arbitrary and may miss some early radiation or focal therapy patients, but we expect these are not large numbers. The data are not population based but are clinically richer, more geographically diverse, and more current than other published series. They therefore provide unique insights into the rapidly evolving landscape of low-risk prostate cancer management.

## Conclusions

This cohort study found that national, community-based rates of AS have increased but remain suboptimal, with wide variation across practices and practitioners. The AQUA Registry has defined the use of AS for low-risk disease as a critical quality indicator for urology. We hope over time to document further reductions in overtreatment rates, in turn improving the benefit-to-harm ratio for early detection and risk-adapted management of prostate cancer.

## Notes

Supplement 1. **eFigure 1.** Locations of AQUA Practices **eFigure 2.** CONSORT-Style Patient Inclusion Diagram **eFigure 3.** Treatment of Low-risk Prostate Cancer Over Time Among Black (Top Panel) and White (Bottom Panel) Patients

Supplement 2.
Data Sharing Statement
