# Supplementary material for: Time Trends and Variation in the Use of Active Surveillance for Management of Low-risk Prostate Cancer in the US
Source: JAMA Netw Open. 2023 Mar 2;6(3):e231439. doi: 10.1001/jamanetworkopen.2023.1439 (PMC9982696; doi:10.1001/jamanetworkopen.2023.1439)
Supplement: Supplement 1. — eFigure 1. Locations of AQUA Practices eFigure 2. CONSORT-Style Patient Inclusion Diagram eFigure 3. Treatment of Low-risk Prostate Cancer Over Time Among Black (Top Panel) and White (Bottom Panel) Patients [file jamanetwopen-e231439-s001.pdf]

Supplementary Online Content

Cooperberg MR, Meeks W, Fang R, Gaylis FD, Catalona WJ, Makarov DV. Time trends and variation in the use of active surveillance for management of low-risk prostate cancer in the US. *JAMA Netw Open*. 2023;6(3):e231439. doi:10.1001/jamanetworkopen.2023.1439

eFigure 1. Locations of AQUA Practices
eFigure 2. CONSORT-Style Patient Inclusion Diagram
eFigure 3. Treatment of Low-risk Prostate Cancer Over Time Among Black (Top Panel) and White (Bottom Panel) Patients

This supplementary material has been provided by the authors to give readers additional information about their work.

© 2023 Cooperberg MR et al. *JAMA Network Open.*

eFigure 1. Locations of AQUA Practices
Circle sizes represent numbers of urology providers per practice.

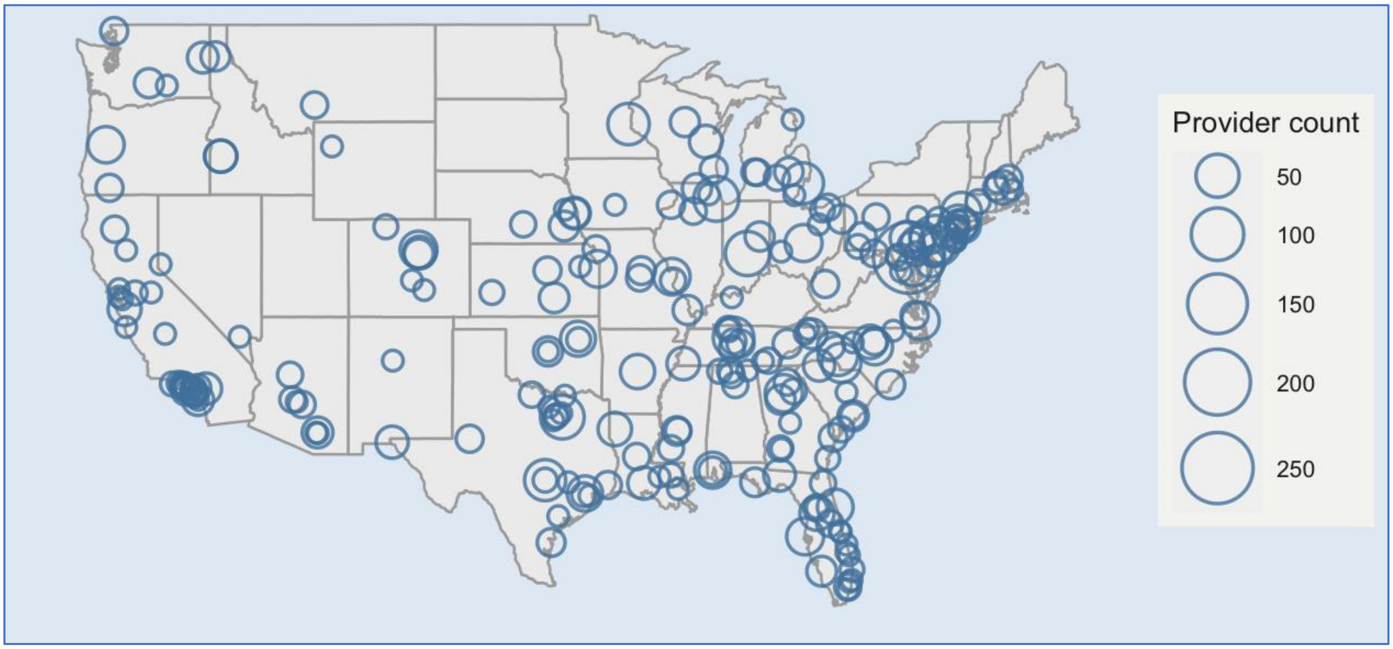

© 2023 Cooperberg MR et al. *JAMA Network Open.*

eFigure 2. CONSORT-Style Patient Inclusion Diagram

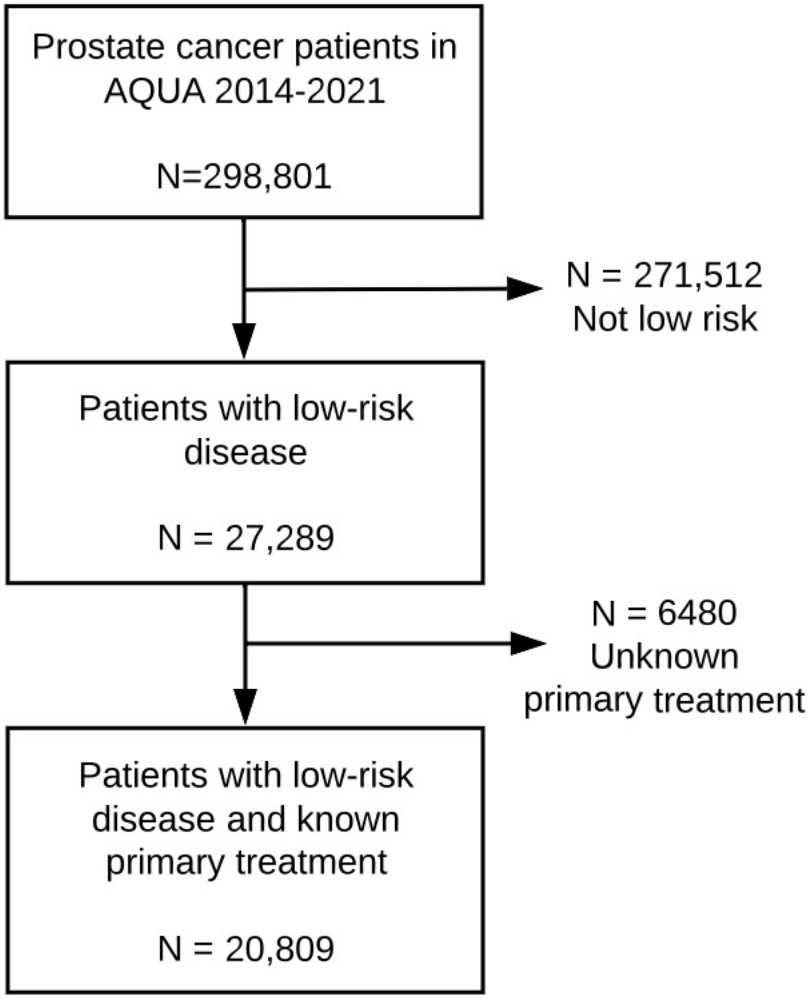

© 2023 Cooperberg MR et al. *JAMA Network Open.*

eFigure 3. Treatment of Low-risk Prostate Cancer Over Time Among Black (Top Panel) and White (Bottom Panel) Patients
ADT = androgen deprivation monotherapy, EBRT = external-beam radiation therapy

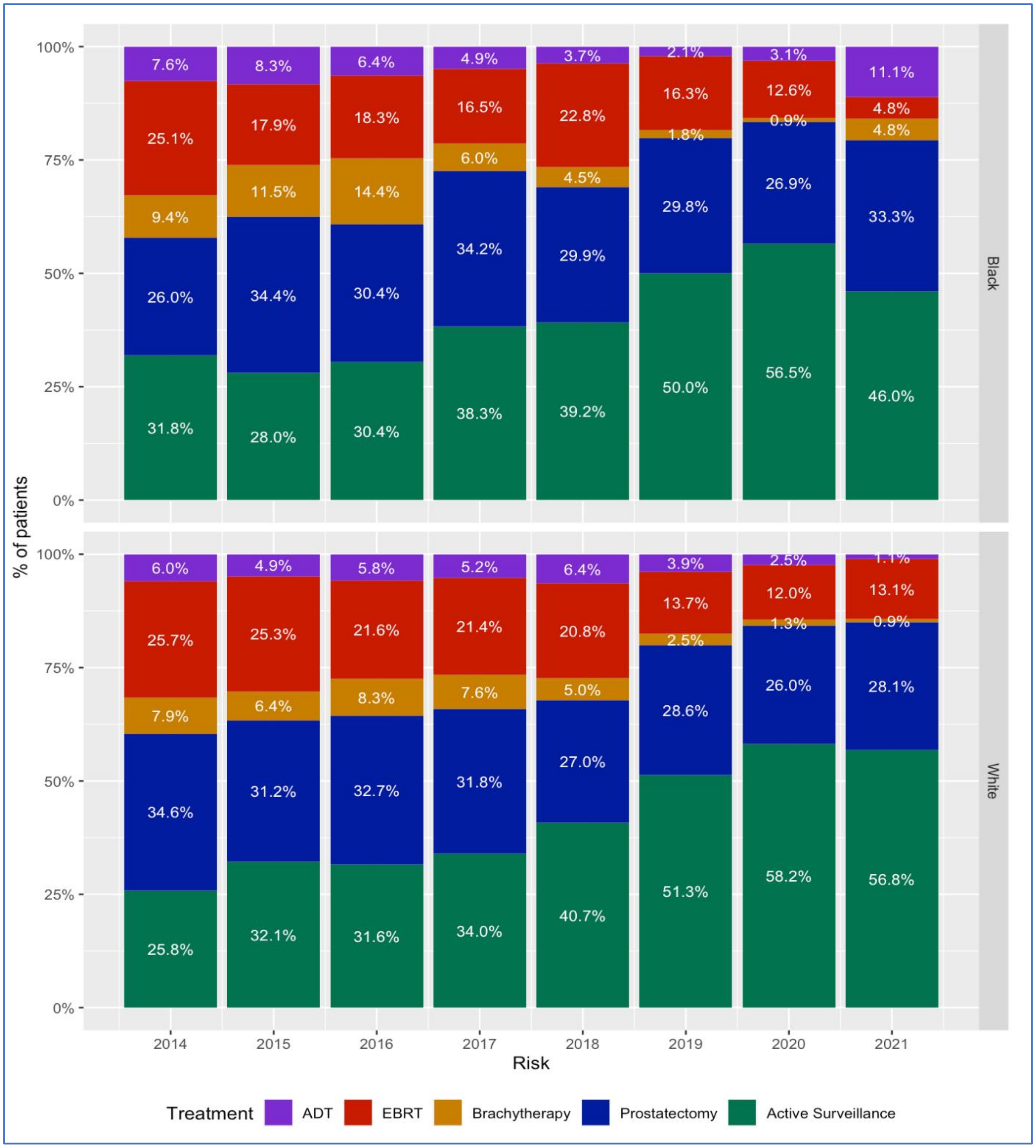

© 2023 Cooperberg MR et al. *JAMA Network Open.*
