# Supplementary material for: Time Trends and Variation in the Use of Active Surveillance for Management of Low-risk Prostate Cancer in the US
Source: JAMA Netw Open. 2023 Mar 2;6(3):e231439. doi: 10.1001/jamanetworkopen.2023.1439 (PMC9982696; doi:10.1001/jamanetworkopen.2023.1439)
Supplement: Supplement 2. — Data Sharing Statement [file jamanetwopen-e231439-s002.pdf]

Data Sharing Statement

Cooperberg. Time Trends and Variation in the Use of Active Surveillance for Management of Low-risk Prostate Cancer in the US. *JAMA Netw Open*. Published March 02, 2023. doi:10.1001/jamanetworkopen.2023.1439

Data
Data available: No

Additional Information
Explanation for why data not available: Participation agreements between urology practices in AQUA and the AUA preclude sharing of patient-level data with 3rd parties without express permission by the practices.
